# Cuproptosis: lipoylated TCA cycle proteins-mediated novel cell death pathway

**DOI:** 10.1038/s41392-022-01014-x

**Published:** 2022-05-13

**Authors:** Su-Ran Li, Lin-Lin Bu, Lulu Cai

**Affiliations:** 1 The State Key Laboratory Breeding Base of Basic Science of Stomatology (Hubei-MOST) & Key Laboratory of Oral Biomedicine Ministry of Education, School and Hospital of Stomatology, Wuhan University, Wuhan, 430072 China; 2 Department of Oral Maxillofacial Head Neck Oncology, School and Hospital of Stomatology, Wuhan University, Wuhan, Hubei 430079 China; 3 Department of Pharmacy, Personalized Drug Therapy Key Laboratory of Sichuan Province, Sichuan Provincial People’s Hospital, School of Medicine, University of Electronic Science and Technology of China, Chengdu, 610072 China

**Keywords:** Senescence, Drug development

In a recent study published in *Science*, Tsvetkov et al^1^. shed a light on a new form of cell death, copper-dependent cell death (termed cuproptosis). They defined “cuproptosis” as a nonapoptotic cell death pathway. They have demonstrated that copper directly binds to lipoylated components of the tricarboxylic acid (TCA) cycle. Then aggregation of these copper-bound, lipoylated mitochondrial proteins and subsequent Fe-S cluster protein loss triggered proteotoxic stress and a distinct form of cell death.

As an essential cofactor, copper homeostasis is critical for various physiological processes. Dysregulation of intracellular copper bioavailability can induce oxidative stress and cytotoxicity^2^. In the animal kingdom, from prokaryotes to eukaryotes, copper homeostasis is finely regulated mainly by preventing excessive accumulation of copper ions in cells which threats cell survival. Pioneering studies have explored several cell death forms, such as apoptosis, necroptosis, pyroptosis, and ferroptosis, however, the phenomenon that copper overload causes cellular toxicity has been less elucidated. In terms of the exact mechanism by which copper ions cause cell death, several hypotheses have been proposed, including the induction of apoptosis, caspase-independent cell death, the induction of reactive oxygen species (ROS), and inhibition of ubiquitin-proteasome system^1^, etc. However, a well-acknowledged theory has not been clearly defined yet. Thus, precise mechanisms of copper-induced cell death still need to be further elucidated.

In this study, Tsvetkov et al. firstly proposed “cuproptosis”, which depended on the accumulation of copper in cells and was a unique cell death pathway distinct from all other known ones. Copper ionophores (like elesclomol) are small molecular transporters of copper ions into cells, which can be excellent tools to explore the mechanisms of cytotoxicity of copper ions. This work illuminated that copper toxicity was highly correlated with mitochondrial activity. Cells with active mitochondrial respiration were more sensitive to elesclomol treatment than cells that rely on anaerobic glycolysis, and the copper accumulation induced cell death with the participation of pivotal components of the TCA cycle.

**Fig. 1:**
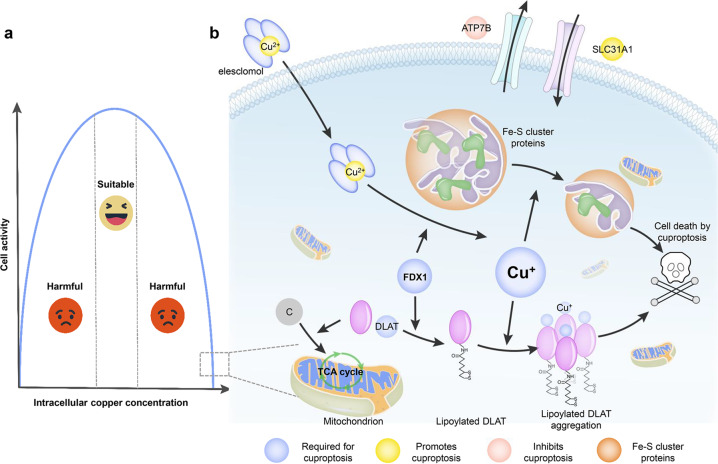
Schematic illustration of cuproptosis mechanism. The effect of intercellular copper concentration on cell activity shows that cells maintain highly bioactive only within a suitable range of extremely narrow copper ion concentrations (**a**). Elesclomol, a copper ionophore, shuttles copper into the cells. FDX1 encodes a reductase to reduce Cu^2+^ to Cu^+^ and is a direct target of elesclomol. DLAT, a protein target of lipoylation, involves with mediating carbon entry to the TCA cycle. DLAT lipoylation was promoted by FDX1, and Cu^+^ enhanced lipoylated protein aggregation and iron-sulfur cluster protein reduction, which triggered proteotoxic stress and cell death (**b**).

To determine the specific metabolic pathway of copper-mediated cytotoxicity, genome-wide CRISPR-Cas9 loss-of-function screens were performed, followed by individual gene knockout studies for further identification of key genes responsible for copper-induced cell death. Authors found that the knockout of FDX1 (Ferredoxin 1, a direct target of copper ionophores) or lipoylated enzymes could rescue cells from copper toxicity. Protein lipoylation is a type of posttranslational modification, which is known to occur only in metabolism-related molecules that involve the initiation process of the TCA cycle. Furthermore, to explore the regulatory relationship among the screened genes, the bioinformatics analysis, human tumor samples immunological analysis and gene knockdown technology were carried out. Results suggested that as an upstream molecule, FDX1 regulated protein lipoylation. FDX1 knockout experiment suggested that FDX1 depletion reduced protein lipoylation of DLAT and DLST (TCA cycle components). Then, the authors demonstrated that DLAT and DLST no longer bound copper after the depletion of FDX1. Naturally, the authors concluded that the lipoyl moiety was necessary for copper to bind. The work also illuminated that copper bound directly to DLAT and led to subsequent oligomerization of DLAT dependent on lipoylation. In addition, the mass spectrometric analysis revealed that the cellular loss of Fe-S cluster proteins after elesclomol treatment was dependent on FDX1 and facilitated by proteotoxic stress. Furthermore, human embryonic kidney 293 T and ABC1 cells overexpressing SLC31A1 (a copper importer) showed a higher cellular sensitivity to copper ions at physiological concentrations. Finally, for in vivo verification of the mechanism of cuproptosis, a mouse model of Wilson’s disease in which ATP7B (a copper exporter) was deleted was used, and the result showed the same cytotoxicity as treatment of copper ionophores. A schematic illustration of the cuproptosis mechanism has been presented at the end of this article (Fig. 1).

Pioneering studies have reported that various metal ions can trigger cell death in independent-apoptosis manners, such as phospholipid peroxidation-triggered ferroptosis^3^. However, the exact mechanism by which excessive copper induces cell death has rarely been reported. The work by Tsvetkov et al. provided an in-depth analysis of the mechanisms of cuproptosis, coupled with its potential pathophysiological functions. Copper, platinum, and iron were widely studied in drug delivery systems development, which showed bright prospects in an array of anticancer purposes^4^. Metal, like copper, and iron, were reported to be cytotoxic, thus care must be taken to rigorously test the effects of metal ions themselves on biological processes. Beyond that, there is a pressing need to explore if excessive accumulation leading to cell damage is a common feature of all metals, especially for some metals commonly used as drug carriers (gold, silicon, etc.).

Additionally, in this work, the authors indicated that mitochondrial glutathione (GSH, a natural intracellular copper chaperone) decelerated copper-dependent cell death via suppressing lipoylation and promoting DLAT oligomerization. Evidence suggested that mitochondrial GSH, as a reductase, reduced oxidative phosphorylation, and depleted mitochondrial GSH increased ROS generation precedes procaspase 3 activation (a critical process of apoptosis)^5^. Therefore, whether a connection can be established between cuproptosis inhibition mediated by GSH and apoptosis-related mechanisms remain to be clarified.

The field of cuproptosis is nonetheless nascent in many ways, thus further studies are urgently needed to be carried out in the following aspects. (1) The molecular mechanisms of copper toxicity in cancers need to be fully fleshed out. Additionally, if cuproptosis evolves towards well-established cell death patterns remains to be further illuminated. (2) Clinical trials of copper-induced cytotoxicity should be based on either the discovery of biomarkers from appropriate patient populations or in-depth knowledge of the molecule’s mechanism of action. (3) Given the differences in the abundance of lipoylated proteins and respiratory patterns in various human tumors, authors suggested that the precise range of concentrations that cause cytotoxicity needs to be determined in various diseases model and even cell types, and then personalized copper-based treatment strategies are reasonable and well-founded.

Taken together, this groundbreaking study unlocked a refreshing cell death pathway. The discovery of “cuproptosis” provides a new avenue for anticancer treatments by fully exploiting the pathophysiological role of copper. Additionally, this study inspires us to further explore the cytotoxicity of other metals. Promisingly, cuproptosis translational medicine might be a potential candidate in clinical applications after a series of safety and efficacy tests, not only for genetic disorders, more importantly, for a variety of human cancers.
